# miR-34a regulates adipogenesis in porcine intramuscular adipocytes by targeting *ACSL4*

**DOI:** 10.1186/s12863-020-0836-7

**Published:** 2020-03-14

**Authors:** Wenwen Wang, Xiuxiu Li, Ning Ding, Jun Teng, Shen Zhang, Qin Zhang, Hui Tang

**Affiliations:** Shandong Provincial Key Laboratory of Animal Biotechnology and Disease Control and Prevention, Shandong Agricultural University, No. 61, Daizong Street, Tai’an City, 271018 Shandong Province China

**Keywords:** IMF, miR-34a, *ACSL4*, Pig

## Abstract

**Background:**

Intramuscular fat (IMF) content is an important factor in porcine meat quality. Previously, we showed that miR-34a was less abundant in liver tissue from pigs with higher backfat thickness, compared to pigs with lower backfat thickness. The purpose of this present study was to explore the role of miR-34a in adipogenesis.

**Result:**

Bioinformatics analysis identified Acyl-CoA synthetase long chain family member 4 (*ACSL4*) as a putative target of miR-34a. Using a luciferase reporter assay, we verified that miR-34a binds the *ACSL4* mRNA at the 3’UTR. To examine the role of the miR-34a-*ACSL4* interaction in IMF deposition in the pig, mRNA and protein expression of the *ACSL4* gene was measured in primary intramuscular preadipocytes transfected with miR-34a mimic and inhibitor. Our results showed that *ACSL4* is expressed throughout the entire differentiation process in pig preadipocytes, similar to the lipogenesis-associated genes *PPARγ* and *aP2.* Transfection with miR-34a mimic reduced lipid droplet formation during adipogenesis, while miR-34a inhibitor increased lipid droplet accumulation. Transfection with miR-34a mimic also reduced the mRNA and protein expression of *ACSL4* and lipogenesis genes, including *PPARγ, aP2,* and *SREBP-1C*, but increased the expression of steatolysis genes such as *ATGL* and *Sirt1*. In contrast, the miR-34a inhibitor had the opposite effect on gene expression. Further, knockdown of *ACSL4* decreased lipid droplet accumulation.

**Conclusions:**

Our results support the hypothesis that miR-34a regulates intramuscular fat deposition in porcine adipocytes by targeting *ACSL4*.

## Background

Intramuscular fat (IMF) content is a primary indicator of porcine meat quality [1]. An increase in IMF content can improve meat flavor [2]. However, substantial efforts have been made to improve production efficiency and select for lean growth, both of which impact IMF negatively. Selection for enhanced IMF has therefore become an important focus in pork production.

High-throughput methods, such as genome-wide association studies and transcriptome expression profiling, have been used to search for genes that potentially affect fat deposition in swine. Many genes associated with an extreme capacity for IMF deposition have been identified [3–9]. In addition to the multiple genes that influence adipogenesis, IMF is also likely to be under the control of post-transcriptional regulatory factors such as microRNAs. MicroRNAs are small non-coding RNA molecules that regulate gene expression by targeting mRNA transcripts for cleavage or translational inhibition [10, 11]. miRNAs play important roles in various biological processes, including cell differentiation, proliferation and apoptosis [12], organ development [13], lipid metabolism [14], and tumorigenesis [15]. Emerging evidence suggests that miRNAs are also involved in adipogenesis. For example, miR-196a induces preadipocyte differentiation by increasing adipocyte marker expression, lipid accumulation, and triglyceride content [16]. miR-27a-5p increases fat deposition in steers partly by targeting the calcium-sensing receptor (CASR) [17]. miR-30e regulates adipocyte differentiation by targeting the low-density lipoprotein receptor-related protein 6 [18]. miR-155, miR-130, and miR-210 inhibit adipocyte formation by targeting the key adipogenic transcriptional factors *PPARγ*, *C/EBPα*, and *TCF712* in the Wnt/β-catenin signaling pathway [19, 20]. We previously identified potential miRNAs regulators of porcine fat deposition by using high-throughput sequencing to examine the transcriptomes in animals with extreme differences in backfat thickness. One of the miRNAs, miR-34a, is markedly less abundant in animals with higher backfat thickness (H group) compared with those with lower backfat thickness (L group) [9]. This result suggests that miR-34a may play important roles in porcine adipogenesis.

MicroRNA-34a has attracted interest recently because of its ability to modulate a myriad of oncogenic functions in different cancers [21–27]. Not only does it play a role in cancer metastasis [28, 29] and drug resistance [30], it is now being evaluated as a diagnostic as well as a prognostic biomarker [31–33]. In addition, a miR-34a inhibitor has been identified that may effectively protect against sevoflurane-induced hippocampal apoptosis by targeting *Wnt1* and activating the Wnt/β-catenin pathway [34]. miR-34a is involved in the pathogenesis of non-alcoholic fatty liver disease [35] and is down-regulated in genetically improved farmed tilapia (*Oreochromis niloticus*) when they are fed a high-fat diet [36]. However, little is known about the role of miR-34a in porcine adipogenesis.

To explore the function of miR-34a in swine, we used bioinformatics analyses to predict its interactions, and conducted experiments to test our predictions using primary preadipocytes. The results provide insight into the ways in which non-coding RNAs affect IMF in pigs.

## Results

### Biological functions of miR-34a based on target analysis

**Fig. 1:**
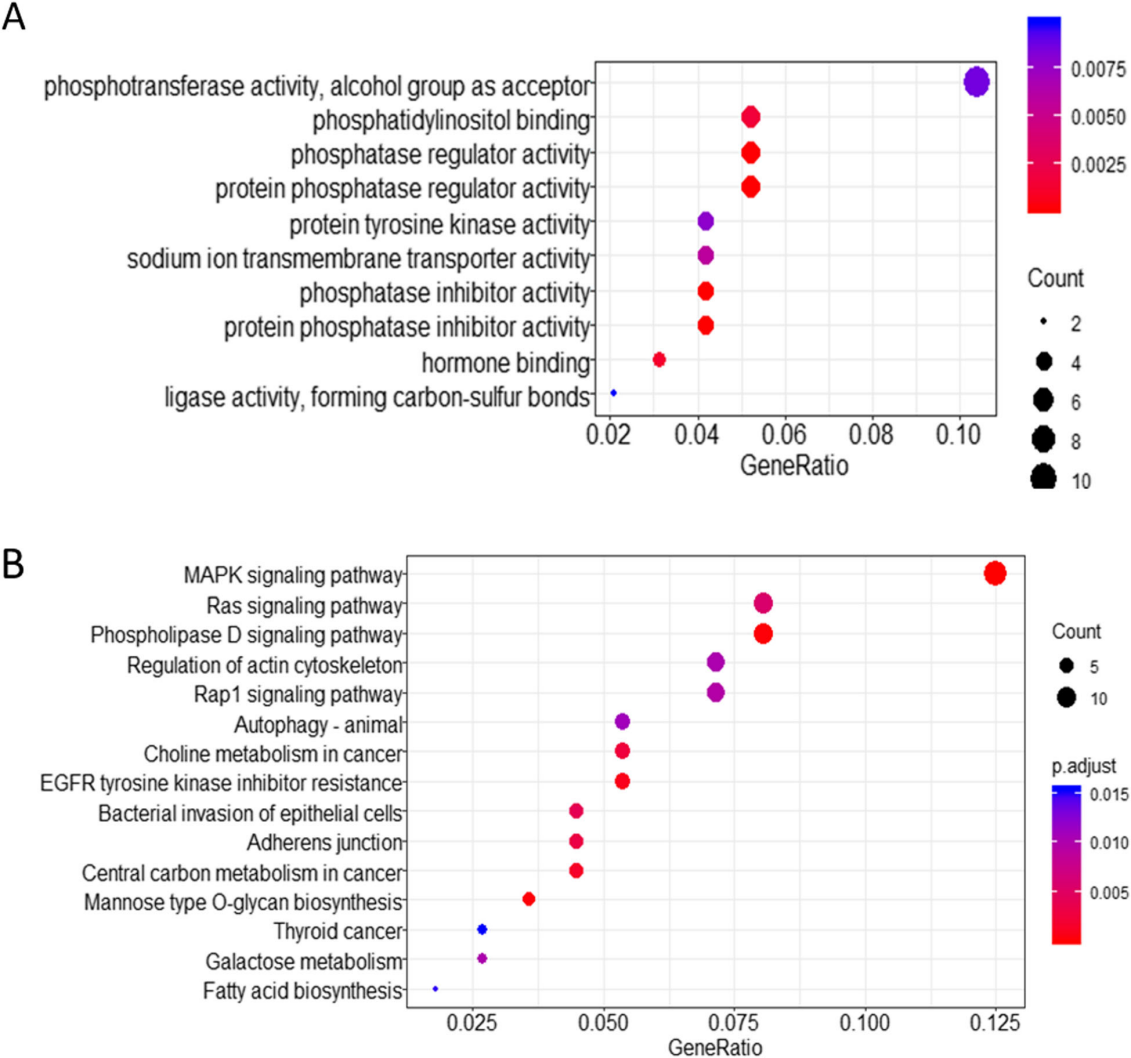
Functional enrichment analysis of predicted miR-34a gene targets. **a** GO categories enriched for genes targeted by miR-34a. **b** KEGG pathways associated with genes targeted by miR-34a

To explore the possible biological functions of miR-34a, the TargetScan and miRDB algorithms were used to predict miR-34a targets. Seven hundred fifty-four and five hundred forty-seven targets were predicted with TargetScan and miRDB, respectively (Supplementary Tables S1 and S2). Two hundred ninety-eight genes overlapped with the targets (Supplementary Table S3), and were examined for potential biological roles using Gene Ontology (GO) term enrichment and Kyoto Encyclopaedia of Genes and Genomes (KEGG) pathway analyses. Several molecular function categories were enriched in the GO analysis (Fig. 1a). Fatty acid biosynthesis and the phosphatidylinositol signaling system were significantly enriched in the KEGG analysis (Fig. 1b).

**Fig. 2:**
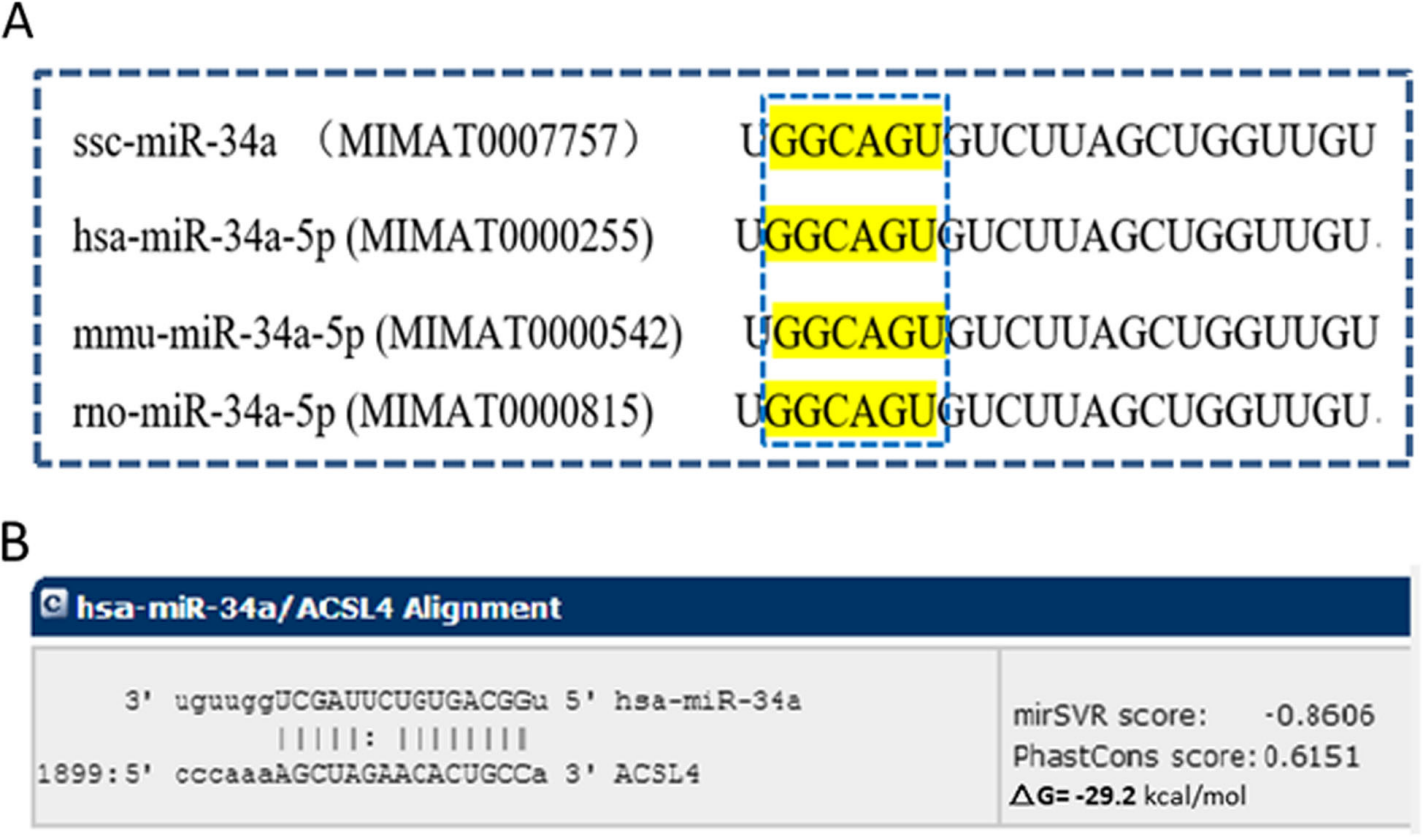
Bioinformatics analysis of miR-34a. **a** Mature sequence of miR-34a is conserved among species including swine (ssc), human (hsa), mouse (mmu), and rat (rno). Data were obtained from miRBase (www.mirbase.org/). **b** Predicted interaction between *ACSL4* 3’UTR and miR-34a

The mature miR-34a sequence is highly conserved in various species, including human, mouse, and rat (Fig. 2a). miRNA target prediction identified *ACSL4* as a potential target gene of miR-34a, with an estimated free energy of − 29.2 kcal/mol for the interaction between them. *ACSL4* encodes acyl-CoA synthetase long chain family member 4, which generates fatty acyl-CoA esters from long-chain fatty acids. The putative target site in the *ACSL4* mRNA is shown in Fig. 2b.

### Interaction between miR-34a and *ACSL4*

**Fig. 3:**
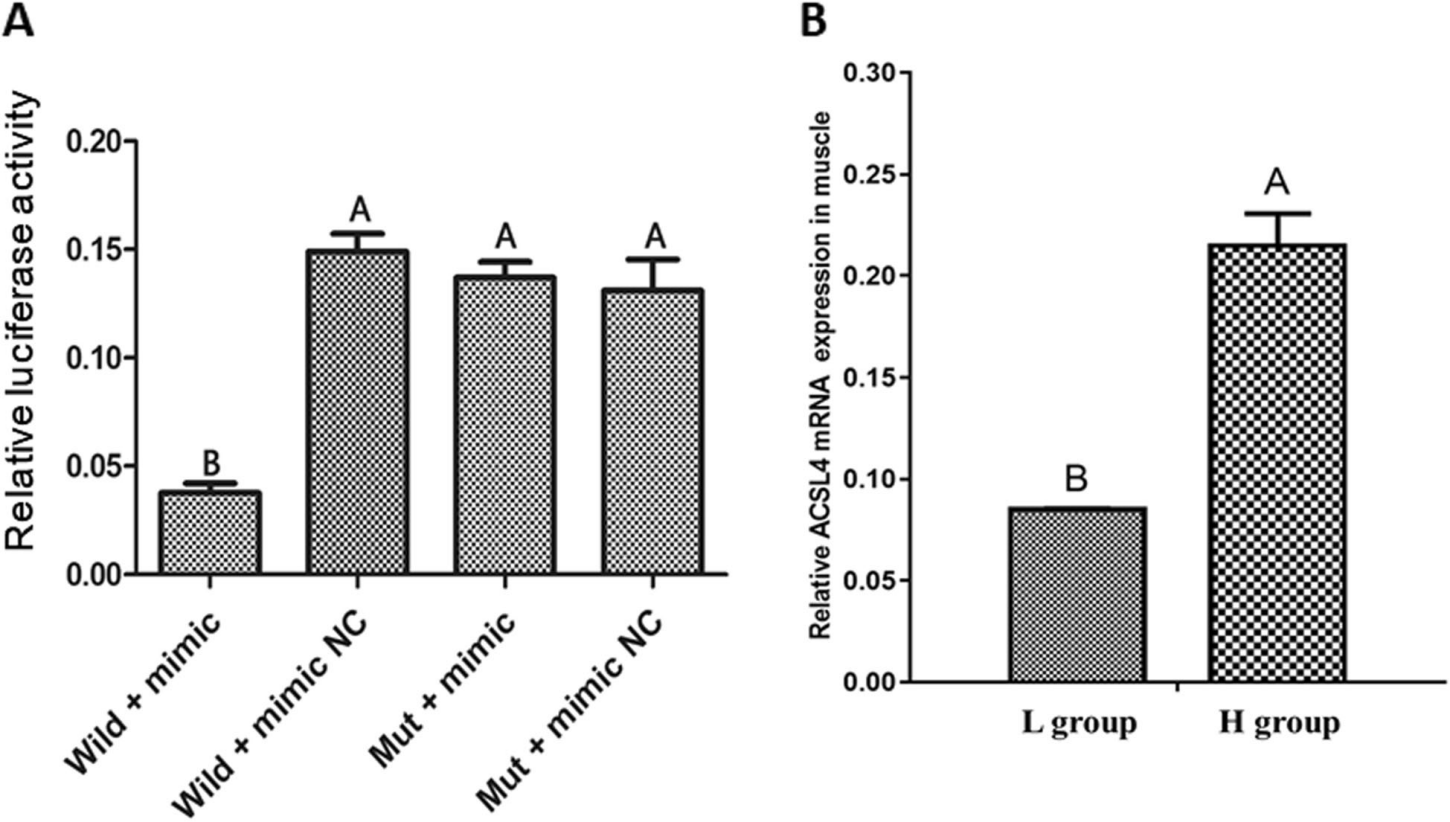
**a** Dual luciferase reporter assay to detect targeting of *ACSL4* by miR-34a in 293 T cells. **b** The relative expression of *ACSL4* mRNA in muscle tissues obtained from animals in the H and L groups. Results are presented as means ± SE of three independent determinations. Labels (A vs. B) indicate significantly different values (*P* < 0.01)

To verify that *ACSL4* is a target of miR-34a, we tested their ability to interact in 293 T cells using a dual-luciferase reporter system. miR-34a mimic significantly decreased luciferase activity generated by the wild-type *ACSL4* reporter vector, compared to the negative control (*P* < 0.01). In contrast, luciferase activity was not affected when a mutated version of the putative miR-34a interaction region was transfected into the cells (Fig. 3a). These results support the hypothesis that *ACSL4* mRNA is targeted by miR-34a. We further detected the expression of *ACSL4* in muscle tissue, which revealed a higher expression in the H group than that in the L group (Fig. 3b).

### Expression of *ACSL4* during porcine preadipocyte differentiation

**Fig. 4:**
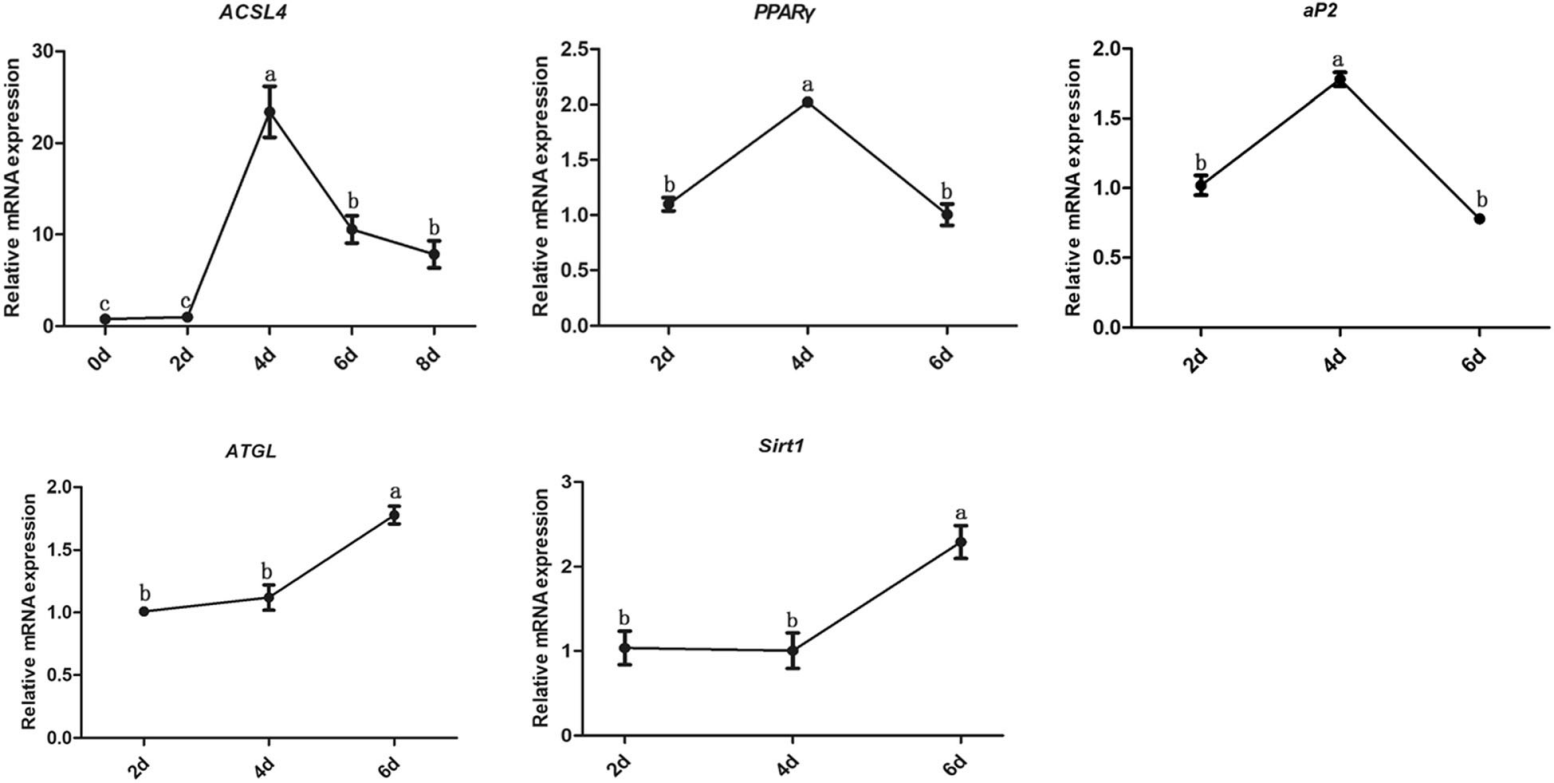
Expression of *ACSL4* and other lipid metabolism-associated genes during preadipocyte differentiation in vitro (0, 2, 4, 6, and 8 days). The results are presented as means ± SE of three independent determinations. Labels (a, b, c) indicate significantly different values (*P* < 0.05)

To examine whether *ACSL4* is a potential contributor to IMF deposition, expression of *ACSL4* was measured by qRT-PCR during preadipocyte differentiation (0, 2, 4, 6, and 8 days after induction). Other marker genes that are widely used in studies of lipid metabolism [37] such as *PPARγ, aP2, ATGL,* and *Sirt1* were also included. As shown in Fig. 4, expression of *ACSL4* mRNA gradually increased after adipocytes were induced to differentiate. Expression peaked at 4 days, the time at which a majority of preadipocytes differentiated into mature adipocytes, and then declined steadily (Fig. 4). Interestingly, similar expression patterns were also observed for lipogenesis transcripts such as *PPARγ* and *aP2* (Fig. 4)*.* In contrast, expression of the steatolysis genes *ATGL* and *Sirt1* increased steadily during preadipocyte differentiation (Fig. 4). The results are consistent with the hypothesis that *ACSL4* is involved in lipogenesis.

### miR-34a inhibits lipogenesis by targeting *ACSL4*

**Fig. 5:**
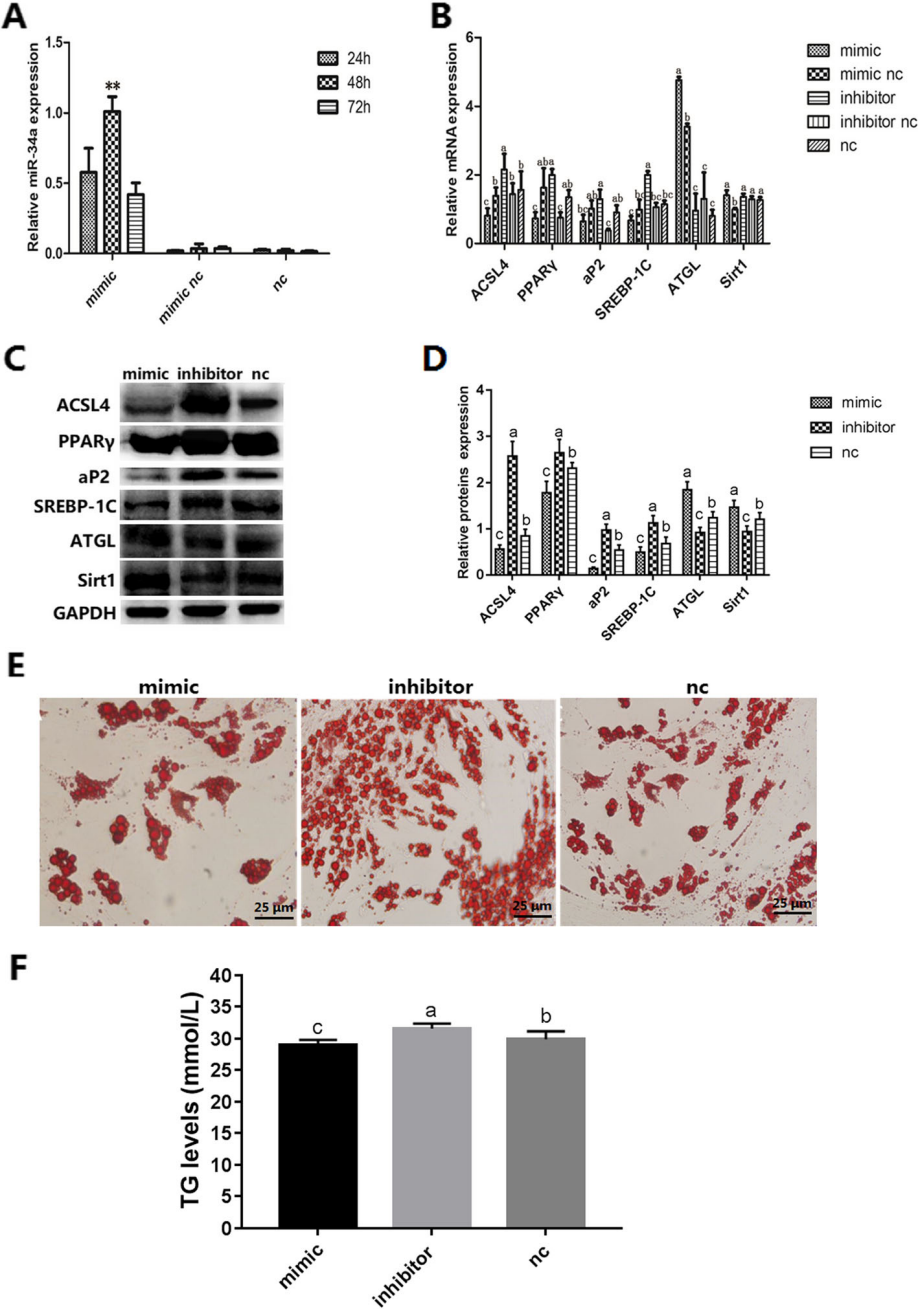
miR-34a inhibits lipogenesis in porcine preadpocytes. **a** Detection of miR-34a mimic after transfection for 24, 48, and 72 h. mimic nc: mimic negative control; nc: blank negative control. **b** Expression of mRNA from genes related to lipogenesis and steatolysis in differentiated cells transfected with miR-34a mimic, mimic negative control, inhibitor, inhibitor negative control, and blank negative control. **c** Proteins detected by western blotting in the mimic, inhibitor, and nc groups. **d** Protein expression in the mimic, inhibitor, and nc groups. **e** Oil Red O staining of terminally differentiated adipocytes. **f** TG levels in the mimic, inhibitor, and nc groups. Results are presented as means ± SE; *n* = 3; ** *P* < 0.01. Labels (a, b, c) indicate significantly different values (*P* < 0.05)

The results of the dual luciferase assay described earlier strongly suggested that miR-34a and *ACSL4* mRNA interact. To test if miR-34a affects lipid metabolism, a mimic and an inhibitor of miR-34a were transfected into porcine preadipocytes. As shown in Fig. 5a, the miR-34a mimic was detected after transfection, with the highest levels observed after 48 h. We then used qRT-PCR and western blotting to measure mRNA and protein expression of *ACSL4* and other genes related to lipid metabolism 48 h after transfection of preadipocytes with miR-34a mimic and inhibitor (Fig. 5b, c, d). As expected, transfection with miR-34a mimic significantly suppressed mRNA and protein expression of *ACSL4* and other lipogenesis genes, including *PPARγ, aP2,* and *SREBP-1C*, and increased expression of steatolysis genes, such as *ATGL* and *Sirt1*. In contrast, miR-34a inhibitor had the opposite effect on the mRNA and protein expression of lipogenesis and steatolysis-related genes, suggesting that miR-34a inhibits lipogenesis by targeting *ACSL4*. Consistent with this result, Oil Red O and triglyceride (TG) quantification assays revealed that the miR-34a mimic significantly decreased lipid droplet numbers, while the miR-34a inhibitor increased them (Fig. 5e, f).

### Silencing of the *ACSL4* gene decreases accumulation of lipid droplets

**Fig. 6:**
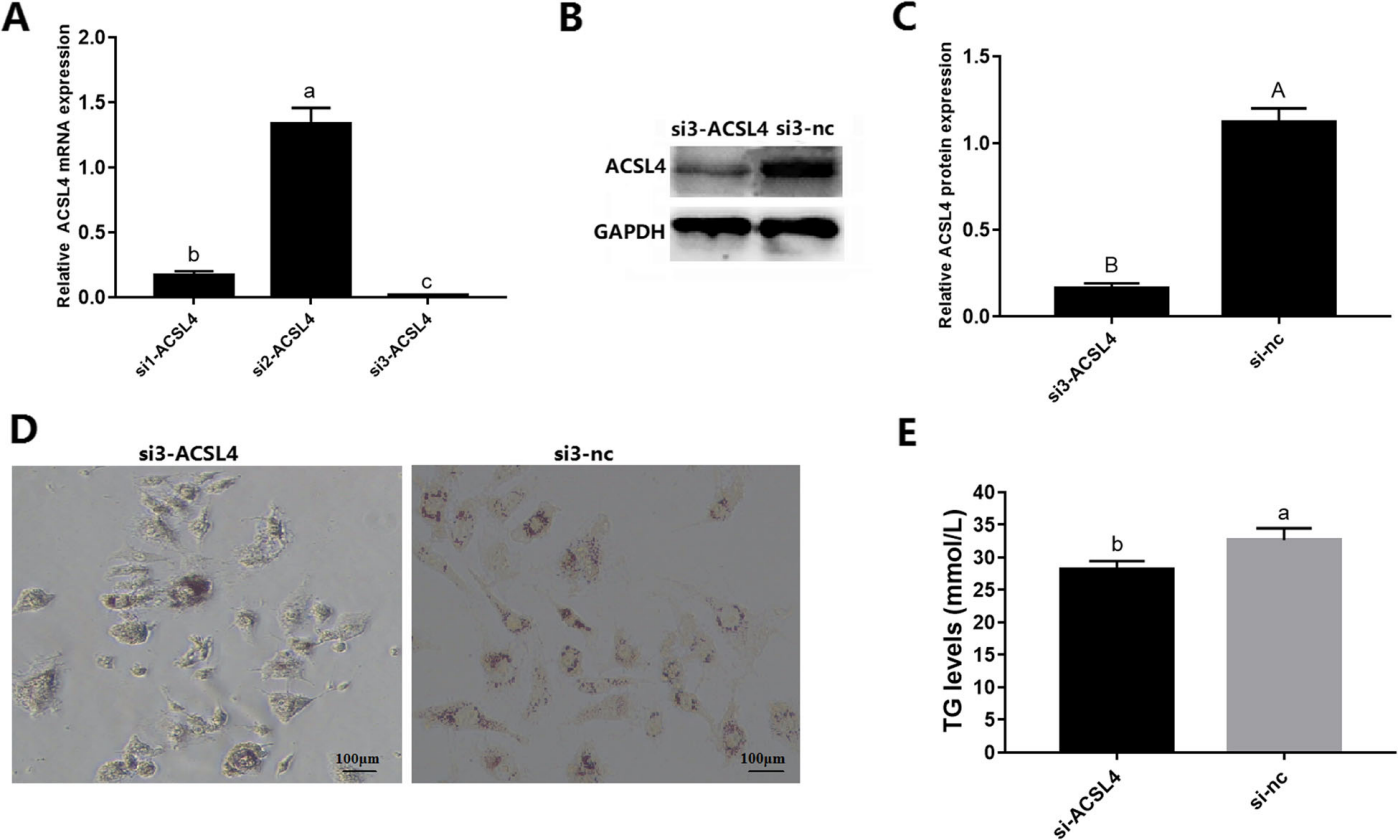
Si-ACSL4 decreased lipid droplet accumulation. **a** Relative mRNA expression levels of *ACSL4* in cells transfected with si1-ACSL4, si2-ACSL4, and si3-ACSL4 fragments. **b** Western blots showing proteins from cells transfected with si3-ACSL4 fragments (GAPDH as the internal reference). **c** ACSL4 protein expression in cells transfected with si3-ACSL4 fragments (GAPDH as the internal reference). **d** Oil Red O staining of terminally differentiated adipocytes. **e** TG levels in the si3-ACSL4 and si3-nc groups. Results are presented as means ± SE; n = 3; Labels (a, b, c) indicate significantly different values (*P* < 0.05). Labels (A vs. B) indicate extremely significantly different values (*P* < 0.01)

RNA interference was used to investigate the function of *ACSL4* in adipogenesis. Three si-ACSL4 fragments (si1-ACSL4, si2-ACSL4, si3-ACSL4) were designed (Supplementary Table S4), and si3-ACSL4 was found to have the highest interference effect (Fig. 6a, b, c). Further, Oil Red O and TG quantification assays showed that knockdown of *ACSL4* reduced lipid droplet accumulation (Fig. 6d, e), similar to the effect of miR-34a mimic (Fig. 5e, f). Taken together, these results demonstrated that miR-34a negatively regulates adipogenesis in porcine adipocytes by targeting *ACSL4*.

## Discussion

Numerous miRNAs regulate adipogenesis by interacting with transcription factors or important signaling molecules that are involved in adipocyte differentiation [38]. Based on our previous studies, miR-34a is less abundant in liver tissue from pigs with higher backfat thickness, compared with pigs with lower backfat thickness [9]. Bioinformatics analysis suggested that potential miR-34a targets functioned in MAPK signaling, regulation of the actin cytoskeleton, galactose metabolism, and fatty acid biosynthesis (Fig. 1). The potential involvement of miR-34a in fatty acid biosynthesis, in combination with the high expression of miR-34a in pigs with low backfat thickness, led us to hypothesize that miR-34a functions in lipid metabolism.

In this study, we investigated the mechanism by which miR-34a affects lipid metabolism in pigs. Sequence analysis suggested that miR-34a targets the *ACSL4* mRNA within the 3’UTR (Fig. 2). In a dual luciferase assay, overexpression of miR-34a inhibited luciferase activity generated by wild-type *ACSL4*, but did not affect activity of a construct containing a mutated version of the putative miR-34a interaction region (Fig. 3a). The porcine *ACSL4* gene is a member of the long-chain acyl-CoA synthetase (ACSL) family of enzymes that catalyze the addition of a coenzyme-A (CoA) group to a fatty acid to form fatty acyl-CoAs [39, 40]. Five ACSL isoforms can each activate and channel various fatty acids to different metabolic fates [40]. Proposed functions of ACSL4 include intracellular lipid storage [41], cholesterol transport from the endoplasmic reticulum into the mitochondria [42], and regulation of arachidonic acid and its metabolites [43–46]. In addition, *ACSL4* polymorphism is associated with IMF content and fatty acid composition in different pig breeds [47–49].

To verify that *ACSL4* plays a role in the regulation of lipid metabolism, we measured the expression of *ACSL4* during porcine preadipocyte differentiation. *ACSL4* mRNA was expressed throughout the entire differentiation process, and abundant in middle term after adipocyte differentiation (Fig. 4). By studying adipocyte differentiation in vitro using various preadipose cell lines and primary preadipocytes, it has been possible to dissect the molecular and cellular events that occur during the transition from undifferentiated fibroblast-like preadipocytes into mature round fat cells [50]. As with primary human, mouse, and rat preadipocyte cell lines, primary pig preadipocytes also proliferate and differentiate, becoming adipocytes with lipid droplets in vitro (Supplementary Figure S1) [51]. We also analyzed the expression of *PPARγ* and *aP2*, which are prominent adipocyte marker genes, and *ATGL* lipases and deacetylase *Sirt1* [52]. Similar expression patterns were observed for *ACSL4* and lipogenesis-associated genes, while the expression patterns for the steatolysis genes were different (Fig. 4). Also, the expression of *ACSL4* mRNA in muscle tissue is higher in the H group than that in the L group (Fig. 3b). This suggests that *ACSL4* is involved in pig lipogenesis.

To further investigate whether interaction between miR-34a and *ACSL4* mRNA plays a role in lipid deposition, miR-34a mimic, inhibitor, and NC were transfected into porcine preadipocytes. As expected, miR-34a mimic decreased the number of lipid droplets, resulting in lower lipid content levels compared to cells transfected with NC. In contrast, miR-34a inhibitor increased the lipid droplet number, resulting in higher lipid content levels (Fig. 5e, f). Our results are consistent with one previous study [53], but are inconsistent with a recent report that overexpression of miR-34a increases lipid deposition in mouse liver and HepG2 cells [54]. The discrepancy may be due to differences between species or cell type. A particular miRNA may play different roles at different developmental stages within one cell type, or at the same developmental stage in different cell types [16]. We also investigated whether miR-34a regulates the expression of lipogenesis- and steatolysis-related genes. As shown in Fig. 5, miRNA-34a mimic decreased the expression of *ACSL4* and lipogenesis genes (*PPARγ*, *aP2* and *SREBP-1C*) and increased expression of steatolysis genes (*ATGL* and *Sirt1*). In contrast, the miR-34a inhibitor had the opposite effect on gene expression. Using RNA interference, we investigated the function of *ACSL4* in adipogenesis. The results showed that knockdown of *ACSL4* reduced lipid droplet accumulation (Fig. 6d, e), similar to the effect of miR-34a mimic (Fig. 5e, f). Taken together, the results demonstrate that miR-34a negatively regulates adipogenesis in porcine adipocytes by targeting *ACSL4*. A checklist for microRNA-target interactions (MTI) is presented in Table 1, following the recommended standards for an MTI report [55].

**Table 1:** Checklist for experimentally validated miRNA-target interaction (MTI)

Item	Results
1. miRNA gene name (Entrez ID)	miR-34a (100316602)
2. Target gene name (Entrez ID)	*ACSL4* (448980)
3. Species name (Species ID)	*Sus scrofa domesticus* (9825)
4. Genomic location of MTI Nucleotide sequence	5'CACTGCC
Location according to Ensembl	X:89761656-89761650
Location within a part of a gene	1934-1940 (location within 3'UTR)
Method for experimental validation	Luciferase reporter assay, qPCR, western blot
Cell lines	293 T cell lines, porcine primary intramuscular preadipocytes
5. Sequence variants within MTI	None
6. Associated phenotype	Adipogenesis

Note:Checklist was prepared according to guidelines for the miRNA target reporting standardization [55]

## Conclusions

In this study, we investigated the mechanism by which miR-34a affects lipid metabolism in pigs. First, we demonstrated that miR-34a binds the *ACSL4* mRNA at the 3’UTR using a luciferase reporter assay. We then showed that transfection of porcine preadipocytes with miR-34a mimic reduced lipid droplet formation, while transfection with miR-34a inhibitor increased the accumulation of lipid droplets. Further, knockdown of *ACSL4* also decreased lipid droplet accumulation. Together, our data support the conclusion that miR-34a negatively regulates lipogenesis in porcine adipocytes by targeting *ACSL4.*

## Methods

### miRNA target gene prediction and functional analyses

miRNA targets were predicted using TargetScan 7.2 (http://www.targetscan.org/) [56] and miRDB (http://mirdb.org) [57]. The free energy of the miR-34a-ACSL4 interaction was calculated using RNAhybrid 2.2 (https://bibiserv.cebitec.uni-bielefeld.de/rnahybrid) [58]. GO term and KEGG enrichment analyses for overlapping target genes were performed using the R package ‘clusterProfiler’ [59], with *p*-values calculated using right-sided hypergeometric tests. Figures were prepared using the R package ‘ggplot2’ [60].

### Dual luciferase reporter assay

The mature miR-34a sequence was retrieved from miRBase (http://www.mirbase.org/). The *ACSL4* 3’UTR wild type (WT) and mutated (MUT) sequences were cloned into pmirGLO vectors using the *SacI* and *SalI* restriction sites. Primers for the luciferase reporter assay are listed in Supplementary Table S5. Two hundred ninety-three T cells in logarithmic growth phase were seeded into the wells of a 96-well plate. Upon reaching 80% confluence, the cells were co-transfected with *ACSL4*–3’UTR-Wild plasmid and miR-34a mimic using Lipofectamine 2000. *ACSL4*–3’UTR-Wild + miR-34a mimic NC, *ACSL4*–3’UTR-Mut + miR-34a mimic, and *ACSL4*–3’UTR-Mut + miR-34a mimic NC were also transfected for comparison. After 48 h, luciferase activity was determined with the dual-luciferase reporter assay system (Promega, USA). All luciferase assays were performed in triplicate and the experiment was performed three times.

### Isolation, culture, differentiation, and transfection of porcine primary intramuscular preadipocytes

Six 7-day old Yorkshire were purchased from the experimental farm at the Chinese of Academy Agricultural Sciences. Animals were humanely euthanized by electrical stunning. The longissimus dorsi muscle (LD) was removed from piglets under sterile conditions. Visible connective tissue was removed, and the remaining tissue was finely minced. Following the protocol described in a previous study [53], preadipocytes were isolated using differential rate adherence by subjecting the tissues to digestion for 2 h with 0.1% type II collagenase. The digested sample was filtered aseptically through a 200 μm nylon mesh filter to isolate cells. The filtered and washed cells were seeded at a density of 2.5 × 10^5^ cells per 35-mm culture dish in DMEM/F12 medium with 10% fetal bovine serum (Hyclone, Logan, UT, USA), supplemented with penicillin (100 U/ mL) and streptomycin (100 U/ mL). The cells were incubated at 37 °C under a water-saturated atmosphere containing 95% air and 5% CO_2_. After 2 h, the dishes were washed with a PBS solution 2–3 times to remove nonadherent cells and to obtain the precursor intramuscular-muscle fat cells. Subsequently, fresh complete culture solution was added and replaced every 2 days. After 2 days, the majority of cells had adhered to the culture dish walls (Supplementary Figure S1A). The number of adherent cells continued to increase with time, and spreading cells had triangular or fusiform shapes. After 8 days, cells had formed a single layer and were morphologically similar to primary cells (Supplementary Figure S1B). To examine IMF deposition and droplet morphology in cultured intramuscular adipocytes, cells were collected 2, 4, 6, and 8 days after induction of differentiation and then stained using Oil Red O. After 2 days of induction, a small number of lipid droplets were detected (Supplementary Figure S1C). The abundance of lipid droplets increased gradually from 4 to 6 days (Supplementary Figure S1D, E), and a large number of lipid droplets were apparent at 8 days (Supplementary Figure S1F). The results showed that the isolated cells were intramuscular preadipocytes.

When the cells reached 80% confluence, they were divided into two groups. The first group was induced to differentiate from preadipocytes to adipocytes. The differentiation medium (AIM; adipocyte-inducing medium) consisted of base medium supplemented with 0.5 mM isobutyl methylxanthine (IBMX, Sigma-Aldrich, St. Louis, MO, USA), 1.0 μM dexamethasone (DEX, Sigma, USA) and 1.0 μg/mL insulin (INS, Sigma). After addition of differentiation medium, cells were collected at 0, 2, 4, 6, and 8 days. The second group was used for overexpression and knockdown experiments. These cells were starved for 12 h in Opti-MEM (Gibco, USA), and then transfected with miR-34a mimic (artificially synthesized miR-34a mimic, 100 nM), mimic negative control (NC, 100 nM), inhibitor (anti-miR-34a, 100 nM), inhibitor NC (100 nM) and si1-ACSL4 (100 nM), si2-ACSL4 (100 nM), si3-ACSL4 (100 nM). ACSL4 siRNA sequences were designed by Shenggong technology Co., LTD. All sequences are listed in Supplementary Table S4. Transfection was conducted with Lipofectamine 2000 (Invitrogen, USA) according to manufacturer’s protocol. Transfected preadipocytes were cultured in 6-well plates for 2 days in the presence of AIM, followed by treatment with insulin alone for 1 more day.

### Oil red O staining

Cells were washed three times with phosphate-buffered saline (PBS) and then fixed in 4% paraformaldehyde for 30 min. The cells were washed twice with deionized water and stained with 60% Oil Red O (solvent: isopropanol, 0.5 g Oil Red powder/100 mL). The cells were protected from light for 30 min, washed three times with PBS, and examined under a microscope.

### Determination of triglyceride

TG levels were quantified using a GPO-PAP enzyme assay (Jiancheng, China). The cells were digested with achromatic trypsin and centrifuged for 10 min at a speed of 1000 rpm. The pellet was washed twice with PBS, and the cells were resuspended at 10^6^/mL with ultrapure water. The cells were disrupted by sonication (3–5 s bursts, 3–5 repetitions) or manually while being chilled in an ice bath. The cells were transferred into 96 well plates and divided into three groups. The blank group (BG) was added into 2.5 μL ddH_2_O and 250 μL working fluid. The standard group (SG) was added into 2.5 μL TG (2.26 mmol/L) and 250 μL working fluid. The experimental group (EG) was added into 2.5 μL cells and 250 μL working fluid. The reactions were mixed and incubated at 37 °C for 10 min. Optical density (absorbance) was measured at 510 nm for each well using a microplate reader. TG (mmol/L) = (OD_EG_-OD_BG_)/(OD_SG_-OD_BG_) * calibrator concentration (mmol/L).

### Quantitative real-time PCR

Total RNA was extracted from harvested cells using TRIzol reagent (TaKaRa, Dalian, China), according to the manufacturer’s instructions. RNA concentration was measured using a NanoDrop 2000 (Thermo, Waltham, MA, USA). cDNA was synthesized using the TaKaRa PrimeScript RT reagent kit with gDNA Eraser. Quantitative PCR was conducted using TaKaRa SYGB Premix EX Taq (Tli RNaseH Plus, CA). For miRNA analysis, the One Step PrimeScript miRNA cDNA Synthesis Kit (TaKaRa) and the SYBR PrimeScriptTM miRNA RT-PCR Kit (TaKaRa) were used. Gene expression was normalized to *β-actin*, and the U6 small RNA was used as the internal reference for miRNA measurements. In real-time quantitative PCR, every reaction was performed in triplicate. Levels were calculated using the relative quantification (2^−ΔΔCt^) method [61]. All primers are listed in Supplementary Table S6.

### Western blotting

Cultured cells were washed two times with PBS, digested with 0.25% trypsin and then centrifuged at 1000 rpm for 15 min. The cells were homogenized in radioimmunoprecipitation assay (PIPA) lysis buffer (Beyotime, Shanghai, China) with phenylmethane sulfonyl fluoride (PMSF, Beyotime, Shanghai, China). Total protein was extracted from the supernatants after centrifugation at 12,000 rpm for 10 min. Protein concentration was determined using a BCA Protein Assay Reagent Kit (Beyotime, Shanghai, China). A protein sample of 50 μg was separated by SDS-PAGE, transferred to polyvinylidene fluoride (PVDF) membranes (Beyotime, Shanghai, China), and sealed overnight in 5% sealant. The membranes were washed three times for 10 min with 10 × TBST, blocked with DifcoTM skim milk for 1 h at room temperature, and then incubated at 4 °C overnight with the following rabbit primary antibodies: anti-ACSL4 polyclonal antibody (1:1000, ABclonal, Wuhan, China), anti-Sirt1 polyclonal antibody (1:1000, ABclonal, Wuhan, China), anti-ATGL polyclonal antibody (1:1000, ABclonal, Wuhan, China), anti-aP2 polyclonal antibody (1:1000, ABclonal, Wuhan, China), anti-SREBP-1c polyclonal antibody (1:1000, ABclonal, Wuhan, China), anti-PPARγ polyclonal antibody (1:1000, ABclonal, Wuhan, China), and anti-GAPDH polyclonal antibody (1:1000, ABclonal, Wuhan, China). After three washes with 10 × TBST, the second antibody (IgG 1:5000, ABclonal, Wuhan, China), conjugated with horseradish peroxidase, was added and the reaction was incubated at 37 °C for 1 h. Binding was detected using an ECL chemiluminescence kit (Beyotime, Shanghai, China). A gel imaging instrument (Vilber Lourmat fusion FX 7 Spectra, France) was used to scan the immunoblots, and an image analysis application (FUSIONCAPT, France) was used to determine the relative density of each band. The results are presented as the ratios of the optical densities of targeted proteins to those of GAPDH.

### Statistical analysis

Statistical analyses were performed using SAS 9.4. All experiments were conducted in triplicate, and results are presented as means ± SE. Multiple comparisons were assessed with a one-way analysis of variance followed by Dunnett’s tests. *P*-values < 0.05 were considered to be statistically significant.

## Supplementary information

**Additional file 1: Supplementary Figure S1.** Identification of porcine primary intramuscular preadipocytes. (A-B) Morphology of primary intramuscular preadipocytes observed under an inverted microscope (× 100) after cultivation for (A) 2 days and (B) 8 days. (C-F) Morphological changes and lipid accumulation in intramuscular adipocytes observed by Oil Red O staining (× 400). Cells were collected at (C) 2, (D) 4, (E) 6, and (F) 8 days after induction of differentiation.

**Additional file 2: Supplementary Table S1.** miR-34a target genes predicted using TargetScan. **Supplementary Table S2.** miR-34a target genes predicted using miRDB. **Supplementary Table S3.** miR-34a target genes predicted both by TargetScan and miRDB. **Supplementary Table S4.** The sequences of small interfering RNAs (siRNAs) specifically targeting *ACSL4*. **Supplementary Table S5.** Primers used for luciferase reporter assay. **Supplementary Table S6.** Primers used for real-time quantitative PCR.

## Data Availability

All data generated or analyzed during this study are included in this published article and its supplementary files.

## Abbreviations

IMF: intramuscular fat
ACSL4: acyl-CoA synthetase long chain family member 4
CASR: calcium-sensing receptor
GO: Gene Ontology
KEGG: Kyoto Encyclopaedia of Genes and Genomes
LD: longissimus dorsi muscle
CoA: coenzyme-A
ACSL: long-chain acyl-CoA synthetase
PBS: phosphate-buffered saline

## References

[1] Fernandez X, Monin G, Talmant A, Mourot J, Lebret B (1999). Influence of intramuscular fat content on the quality of pig meat - 1. Composition of the lipid fraction and sensory characteristics of m. longissimus lumborum. Meat Sci.

[2] Hocquette JF, Gondret F, Baeza E, Medale F, Jurie C, Pethick DW (2010). Intramuscular fat content in meat-producing animals: development, genetic and nutritional control, and identification of putative markers. Animal.

[3] Davoli R, Luise D, Mingazzini V, Zambonelli P, Braglia S, Serra A, Russo V (2016). Genome-wide study on intramuscular fat in Italian large white pig breed using the PorcineSNP60 BeadChip. J Anim Breed Genet.

[4] Puig-Oliveras A, Revilla M, Castello A, Fernandez AI, Folch JM, Ballester M (2016). Expression-based GWAS identifies variants, gene interactions and key regulators affecting intramuscular fatty acid content and composition in porcine meat. Sci Rep.

[5] Munoz M, Rodriguez MC, Alves E, Folch JM, Ibanez-Escriche N, Silio L, Fernandez AI (2013). Genome-wide analysis of porcine backfat and intramuscular fat fatty acid composition using high-density genotyping and expression data. BMC Genomics.

[6] Wang Y, Ning C, Wang C, Guo J, Wang J, Wu Y. Genome-wide association study for intramuscular fat content in Chinese Lulai black pigs. Asian-Australas J Anim Sci. 2019;32(5):607–13. 10.5713/ajas.18.0483 PMC6502724 30381738

[7] Ros-Freixedes R, Gol S, Pena RN, Tor M, Ibanez-Escriche N, Dekkers JC, Estany J (2016). Genome-wide association study singles out SCD and LEPR as the two Main loci influencing intramuscular fat content and fatty acid composition in Duroc pigs. PLoS One.

[8] Won S, Jung J, Park E, Kim H (2018). Identification of genes related to intramuscular fat content of pigs using genome-wide association study. Asian-Australas J Anim Sci.

[9] Li W, Yang Y, Liu Y, Liu S, Li X, Wang Y, Zhang Y, Tang H, Zhou R, Li K (2017). Integrated analysis of mRNA and miRNA expression profiles in livers of Yimeng black pigs with extreme phenotypes for backfat thickness. Oncotarget.

[10] Bartel DP (2004). MicroRNAs: genomics, biogenesis, mechanism, and function. Cell.

[11] He L, Hannon GJ (2004). MicroRNAs: small RNAs with a big role in gene regulation. Nat Rev Genet.

[12] Tufekci KU, Meuwissen RL, Genc S (2014). The role of microRNAs in biological processes. Methods Mol Biol.

[13] Bhaskaran M, Mohan M (2014). MicroRNAs: history, biogenesis, and their evolving role in animal development and disease. Vet Pathol.

[14] Novak J, Bienertova-Vasku J, Kara T, Novak M (2014). MicroRNAs involved in the lipid metabolism and their possible implications for atherosclerosis development and treatment. Mediat Inflamm.

[15] Di Leva G, Briskin D, Croce CM (2012). MicroRNA in cancer: new hopes for antineoplastic chemotherapy. Ups J Med Sci.

[16] Ning X, Liu S, Qiu Y, Li G, Li Y, Li M, Yang G. Expression Profiles and Biological Roles of miR-196a in Swine. Genes (Basel). 2016;7(2):5. 10.3390/genes7020005 PMC4773749 26805888

[17] Yang W, Tang K, Wang Y, Zan L (2018). MiR-27a-5p increases steer fat deposition partly by targeting calcium-sensing receptor (CASR). Sci Rep.

[18] Wang J, Guan X, Guo F, Zhou J, Chang A, Sun B, Cai Y, Ma Z, Dai C, Li X (2013). miR-30e reciprocally regulates the differentiation of adipocytes and osteoblasts by directly targeting low-density lipoprotein receptor-related protein 6. Cell Death Dis.

[19] Lee EK, Lee MJ, Abdelmohsen K, Kim W, Kim MM, Srikantan S, Martindale JL, Hutchison ER, Kim HH, Marasa BS (2011). miR-130 suppresses adipogenesis by inhibiting peroxisome proliferator-activated receptor gamma expression. Mol Cell Biol.

[20] Chen Y, Siegel F, Kipschull S, Haas B, Frohlich H, Meister G, Pfeifer A (2013). miR-155 regulates differentiation of brown and beige adipocytes via a bistable circuit. Nat Commun.

[21] Wen D, Peng Y, Lin F, Singh RK, Mahato RI (2017). Micellar delivery of miR-34a modulator Rubone and paclitaxel in resistant prostate Cancer. Cancer Res.

[22] Ito Y, Inoue A, Seers T, Hato Y, Igarashi A, Toyama T, Taganov KD, Boldin MP, Asahara H (2017). Identification of targets of tumor suppressor microRNA-34a using a reporter library system. Proc Natl Acad Sci U S A.

[23] Shi Y, Liu C, Liu X, Tang DG, Wang J (2014). The microRNA miR-34a inhibits non-small cell lung cancer (NSCLC) growth and the CD44hi stem-like NSCLC cells. PLoS One.

[24] Adams BD, Parsons C, Slack FJ (2016). The tumor-suppressive and potential therapeutic functions of miR-34a in epithelial carcinomas. Expert Opin Ther Targets.

[25] Saito Y, Nakaoka T, Saito H (2015). microRNA-34a as a therapeutic agent against human Cancer. J Clin Med.

[26] Li L (2014). Regulatory mechanisms and clinical perspectives of miR-34a in cancer. J Cancer Res Ther.

[27] Li XJ, Ren ZJ, Tang JH (2014). MicroRNA-34a: a potential therapeutic target in human cancer. Cell Death Dis.

[28] Maroni P, Puglisi R, Mattia G, Care A, Matteucci E, Bendinelli P, Desiderio MA (2017). In bone metastasis miR-34a-5p absence inversely correlates with met expression, while met oncogene is unaffected by miR-34a-5p in non-metastatic and metastatic breast carcinomas. Carcinogenesis.

[29] Xiang ZL, Zhao XM, Zhang L, Yang P, Fan J, Tang ZY, Zeng ZC (2016). MicroRNA-34a expression levels in serum and intratumoral tissue can predict bone metastasis in patients with hepatocellular carcinoma. Oncotarget.

[30] Ghandadi M, Sahebkar A (2016). MicroRNA-34a and its target genes: key factors in cancer multidrug resistance. Curr Pharm Des.

[31] Rapti SM, Kontos CK, Christodoulou S, Papadopoulos IN, Scorilas A (2017). miR-34a overexpression predicts poor prognostic outcome in colorectal adenocarcinoma, independently of clinicopathological factors with established prognostic value. Clin Biochem.

[32] Imani S, Zhang X, Hosseinifard H, Fu S, Fu J (2017). The diagnostic role of microRNA-34a in breast cancer: a systematic review and meta-analysis. Oncotarget.

[33] Chen AH, Qin YE, Tang WF, Tao J, Song HM, Zuo M (2017). MiR-34a and miR-206 act as novel prognostic and therapy biomarkers in cervical cancer. Cancer Cell Int.

[34] Zhao X, Sun Y, Ding Y, Zhang J, Li K (2018). miR-34a inhibitor may effectively protect against Sevoflurane-induced hippocampal apoptosis through the Wnt/beta-catenin pathway by targeting Wnt1. Yonsei Med J.

[35] Torres JL, Novo-Veleiro I, Manzanedo L, Alvela-Suarez L, Macias R, Laso FJ, Marcos M (2018). Role of microRNAs in alcohol-induced liver disorders and non-alcoholic fatty liver disease. World J Gastroenterol.

[36] Tao YF, Qiang J, Bao JW, Chen DJ, Yin GJ, Xu P, Zhu HJ (2018). Changes in physiological parameters, lipid metabolism, and expression of MicroRNAs in genetically improved farmed Tilapia (Oreochromis niloticus) with fatty liver induced by a high-fat diet. Front Physiol.

[37] Xu Y, Du J, Zhang P, Zhao X, Li Q, Jiang A, Jiang D, Tang G, Jiang Y, Wang J, et al. MicroRNA-125a-5p Mediates 3T3-L1 Preadipocyte Proliferation and Differentiation. Molecules. 2018;23(2):317. 10.3390/molecules23020317 PMC6017839 29393878

[38] Kim C, Lee H, Cho YM, Kwon OJ, Kim W, Lee EK. TNFalpha-induced miR-130 resulted in adipocyte dysfunction during obesity-related inflammation. FEBS Lett. 2013;S0014-5793(13):00775–8. 24396868

[39] Mashek DG, Li LO, Coleman RA (2007). Long-chain acyl-CoA synthetases and fatty acid channeling. Future Lipidol.

[40] Li LO, Mashek DG, An J, Doughman SD, Newgard CB, Coleman RA (2006). Overexpression of rat long chain acyl-coa synthetase 1 alters fatty acid metabolism in rat primary hepatocytes. J Biol Chem.

[41] Xu X, Gopalacharyulu P, Seppanen-Laakso T, Ruskeepaa AL, Aye CC, Carson BP, Mora S, Oresic M, Teleman AA (2012). Insulin signaling regulates fatty acid catabolism at the level of CoA activation. PLoS Genet.

[42] Duarte A, Poderoso C, Cooke M, Soria G, Cornejo MF, Gottifredi V, Podesta EJ (2012). Mitochondrial fusion is essential for steroid biosynthesis. PLoS One.

[43] Maloberti PM, Duarte AB, Orlando UD, Pasqualini ME, Solano AR, Lopez-Otin C, Podesta EJ (2010). Functional interaction between acyl-CoA synthetase 4, lipooxygenases and cyclooxygenase-2 in the aggressive phenotype of breast cancer cells. PLoS One.

[44] Kuwata H, Hara S (2015). Inhibition of long-chain acyl-CoA synthetase 4 facilitates production of 5, 11-dihydroxyeicosatetraenoic acid via the cyclooxygenase-2 pathway. Biochem Biophys Res Commun.

[45] Kang MJ, Fujino T, Sasano H, Minekura H, Yabuki N, Nagura H, Iijima H, Yamamoto TT (1997). A novel arachidonate-preferring acyl-CoA synthetase is present in steroidogenic cells of the rat adrenal, ovary, and testis. Proc Natl Acad Sci U S A.

[46] Klett EL, Chen S, Edin ML, Li LO, Ilkayeva O, Zeldin DC, Newgard CB, Coleman RA (2013). Diminished acyl-CoA synthetase isoform 4 activity in INS 832/13 cells reduces cellular epoxyeicosatrienoic acid levels and results in impaired glucose-stimulated insulin secretion. J Biol Chem.

[47] Mercade A, Estelle J, Perez-Enciso M, Varona L, Silio L, Noguera JL, Sanchez A, Folch JM (2006). Characterization of the porcine acyl-CoA synthetase long-chain 4 gene and its association with growth and meat quality traits. Anim Genet.

[48] Rusc A, Sieczkowska H, Krzecio E, Antosik K, Zybert A, Kocwin-Podsiadla M, Kaminski S (2011). The association between acyl-CoA synthetase (ACSL4) polymorphism and intramuscular fat content in (landrace x Yorkshire) x Duroc pigs. Meat Sci.

[49] Chen JN, Jiang YZ, Cen WM, Xing SH, Zhu L, Tang GQ, Li MZ, Jiang AA, Lou PE, Wen AX (2014). Distribution of H-FABP and ACSL4 gene polymorphisms and their associations with intramuscular fat content and backfat thickness in different pig populations. Genet Mol Res.

[50] Gregoire FM, Smas CM, Sul HS (1998). Understanding adipocyte differentiation. Physiol Rev.

[51] Morrison RF, Farmer SR (2000). Hormonal signaling and transcriptional control of adipocyte differentiation. J Nutr.

[52] Lasa A, Churruca I, Eseberri I, Andres-Lacueva C, Portillo MP (2012). Delipidating effect of resveratrol metabolites in 3T3-L1 adipocytes. Mol Nutr Food Res.

[53] Sun YM, Qin J, Liu SG, Cai R, Chen XC, Wang XM, Pang WJ. PDGFRalpha Regulated by miR-34a and FoxO1 Promotes Adipogenesis in Porcine Intramuscular Preadipocytes through Erk Signaling Pathway. Int J Mol Sci. 2017;18(11):2424. 10.3390/ijms18112424 PMC5713392 29140299

[54] Wen F, An C, Wu X, Yang Y, Xu J, Liu Y, Wang C, Nie L, Fang H, Yang Z (2018). MiR-34a regulates mitochondrial content and fat ectopic deposition induced by resistin through the AMPK/PPARalpha pathway in HepG2 cells. Int J Biochem Cell Biol.

[55] Piletič K, Kunej T (2017). Minimal standards for reporting microRNA: target interactions. Omics.

[56] Agarwal V, Bell GW, Nam J, Bartel DP (2015). Predicting effective microRNA target sites in mammalian mRNAs. eLife.

[57] Liu W, Wang X (2019). Prediction of functional microRNA targets by integrative modeling of microRNA binding and target expression data. Genome Biol.

[58] Rehmsmeier M, Steffen P, Hochsmann M, Giegerich R (2004). Fast and effective prediction of microRNA/target duplexes. RNA.

[59] Yu G, Wang LG, Han Y, He QY (2012). clusterProfiler: an R package for comparing biological themes among gene clusters. OMICS.

[60] Wilkinson L (2011). ggplot2: elegant graphics for data analysis by H. WICKHAM. Biometrics.

[61] Livak KJ, Schmittgen TD (2001). Analysis of relative gene expression data using real-time quantitative PCR and the 2(−Delta Delta C(T)) method. Methods.

