# Supplementary figures and images for: miR-34a regulates adipogenesis in porcine intramuscular adipocytes by targeting ACSL4
Source: BMC Genet. 2020 Mar 14;21:33. doi: 10.1186/s12863-020-0836-7 (PMC7073017; doi:10.1186/s12863-020-0836-7)

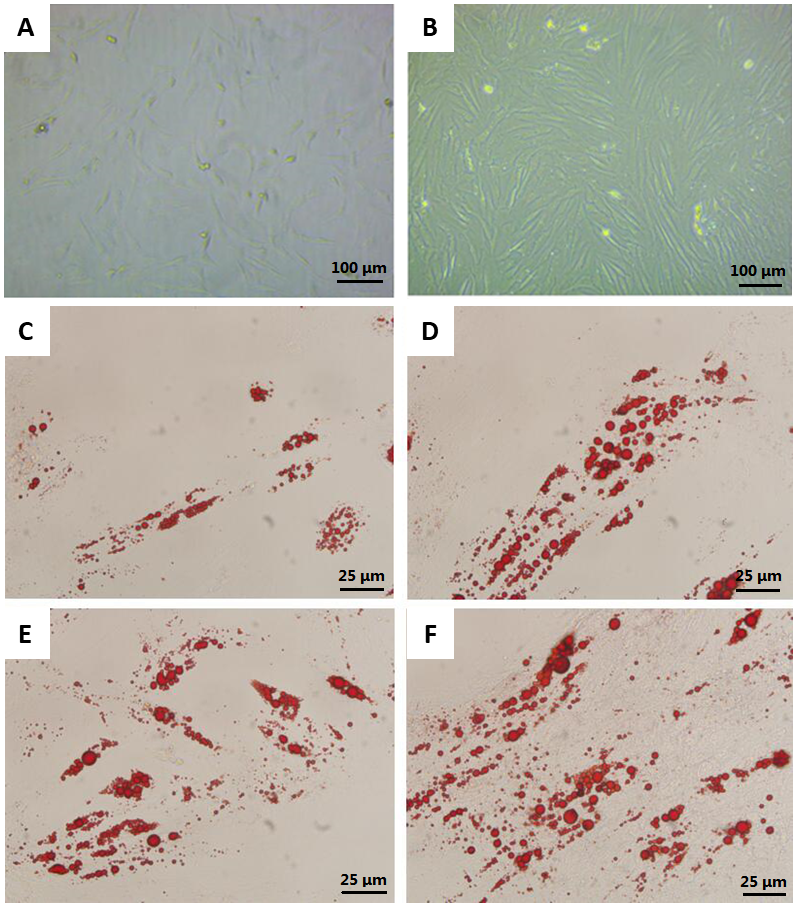

Supplement: Supplementary file 1 — Additional file 1: Supplementary Figure S1. Identification of porcine primary intramuscular preadipocytes. (A-B) Morphology of primary intramuscular preadipocytes observed under an inverted microscope (× 100) after cultivation for (A) 2 days and (B) 8 days. (C-F) Morphological changes and lipid accumulation in intramuscular adipocytes observed by Oil Red O staining (× 400). Cells were collected at (C) 2, (D) 4, (E) 6, and (F) 8 days after induction of differentiation. [file 12863_2020_836_MOESM1_ESM.png]
